# Supplementary material for: Large Igneous Province thermogenic greenhouse gas flux could have initiated Paleocene-Eocene Thermal Maximum climate change
Source: Nat Commun. 2019 Dec 5;10:5547. doi: 10.1038/s41467-019-12957-1 (PMC6895149; doi:10.1038/s41467-019-12957-1)
Supplement: Supplementary file 1 — Supplementary Information [file 41467_2019_12957_MOESM1_ESM.pdf]

Supplementary Information

Large Igneous Province thermogenic greenhouse gas flux could have initiated Paleocene-Eocene Thermal Maximum climate change

S. M. Jones et al.

Contents of this file

Supplementary Figures 1 to 6.

Supplementary Tables 1 and 2.

Other supplementary information available online

Supplementary Movie 1.

Supplementary Figures

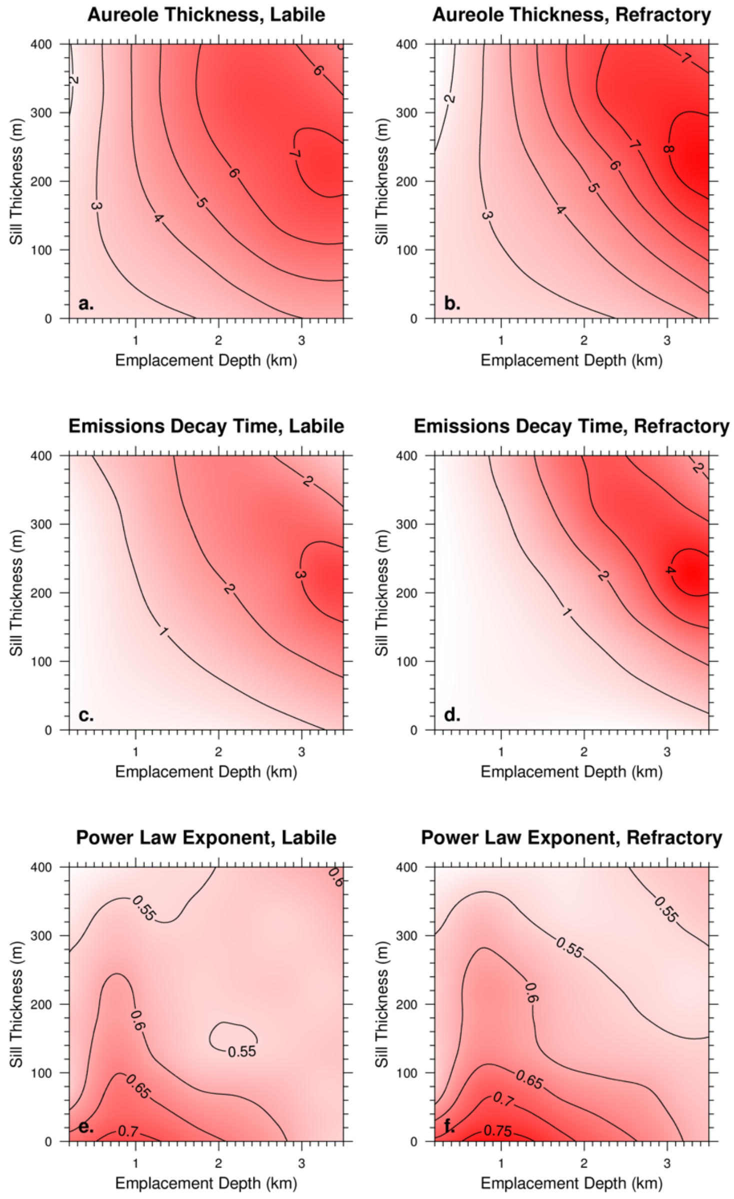

Supplementary Fig. 1. Dependence of carbon emissions parameterisation on sill thickness and emplacement depth.

a. Total thickness Y_{crack} of the reaction aureoles above and below the sill for generation of gas by breakdown of labile kerogen, expressed in multiples of sill thickness. Calculations assume that 20% of kerogen matures directly to gas, and the remaining 80% first matures to oil which then cracks to gas[31]. **b.** Aureole thickness Y_{ref} for breakdown of refractory kerogen, all of which matures directly to gas. **c.** Time scale τ_{decay} for decay of gas emissions from breakdown of labile kerogen, expressed in multiples of the thermal conduction time scale $\tau_{cond} = S^2/\kappa$, where S is the sill thickness and $\kappa = 10^6$ m^2 s^{-1} is the thermal diffusivity of the host rock. **d.** Time scale τ_{decay} for decay of gas emissions from breakdown of refractory kerogen, similarly labelled. **e, f.** Power law exponent p for the breakdown on labile and refractory kerogen respectively.

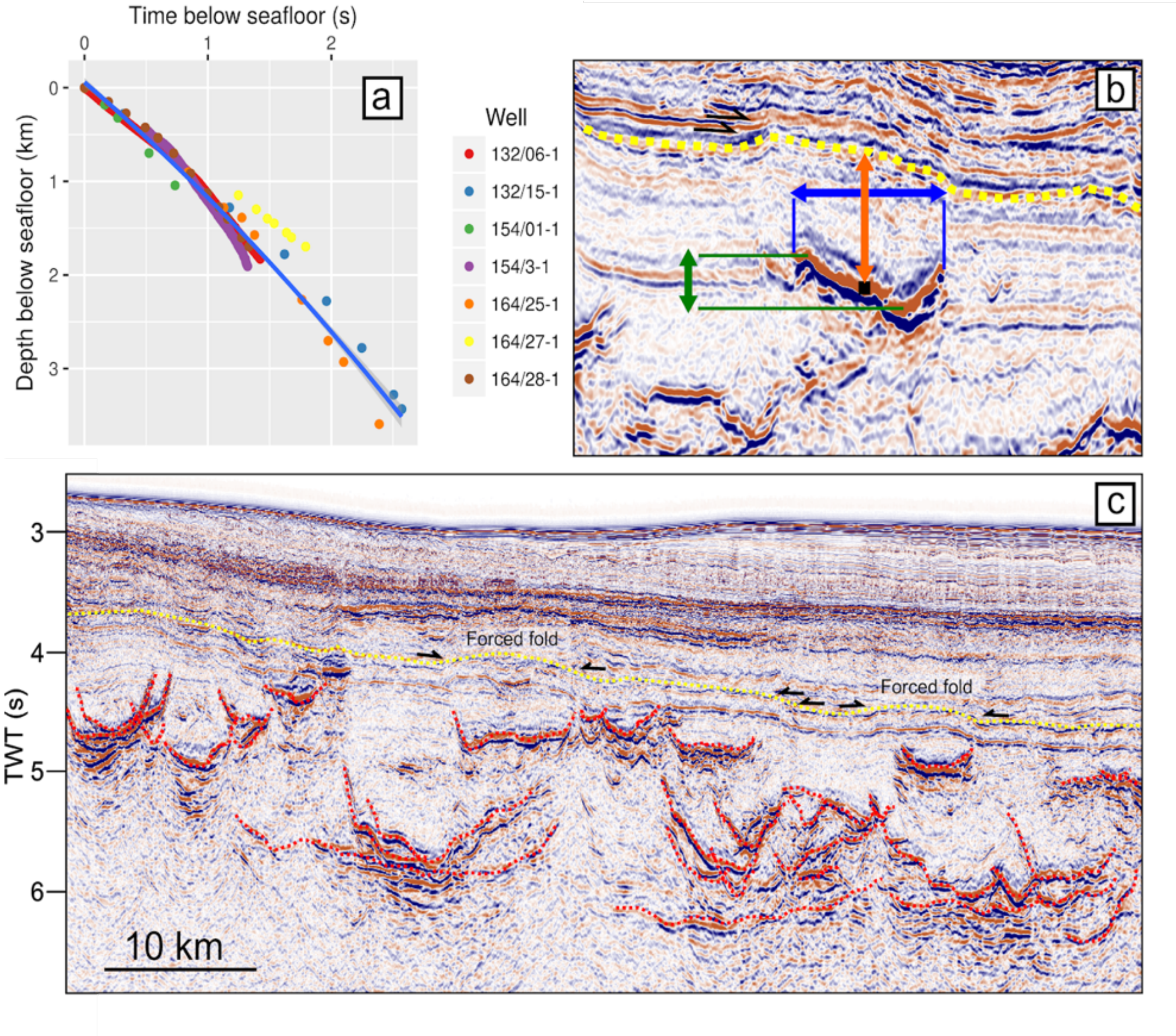

Supplementary Fig. 2. Example of seismic data used to measure sill dimensions.

a. Checkshot surveys from industry wells in Rockall Basin used to depth-covert the measurements. Blue line is the best-fit second order polynomial, used for depth conversion. **b.** Definition of measurements: blue, sill diameter; orange, sill emplacement depth (yellow dashed line is palaeo-seafloor); green, transgressive height. **c.** Example 2D seismic line across part of northern Rockall Basin from a publicly available UK Oil and Gas Authority survey (see ref. [54] for how to access data). Sills are in red and palaeo-seafloor is in yellow. Black arrows indicate onlap onto forced folds that identify the seabed at the time of sill intrusion. Note that all sills (where timing information is available) intruded coeval with the yellow dashed horizon at seismic imaging resolution.

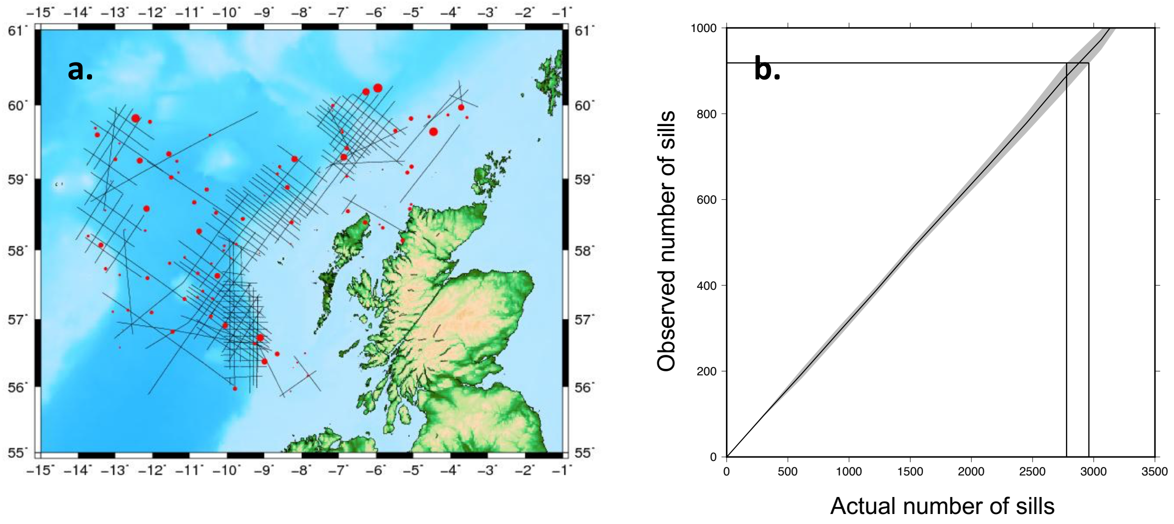

Supplementary Fig. 3. The "Missing sills test" to determine sill intrusion density from 2D seismic surveys.

a. One out of 1200 runs of the Monte Carlo missing sills test for the OGA 2D seismic survey. The survey geometry (lines) is overlain by a synthetic population of sills (red dots) randomly distributed across the survey footprint and with surface areas drawn from the observed distribution. **b.** Number of sills that intersect one or more seismic lines as a function of number of synthetic sills in the population, including uncertainty range obtained from Monte Carlo modelling. The estimated total number of sills is found to be a linear function of the number of sills observed to intersect the 2D grid. The slope of this correlation depends on the surface area distribution of the sill population and the geometry of the seismic grid, so it is different for each 2D survey. The final estimate for the real number of sills is found by projecting horizontally from the observed number of sills (920 in this case) to the line resulting from Monte Carlo modelling, and then vertically.

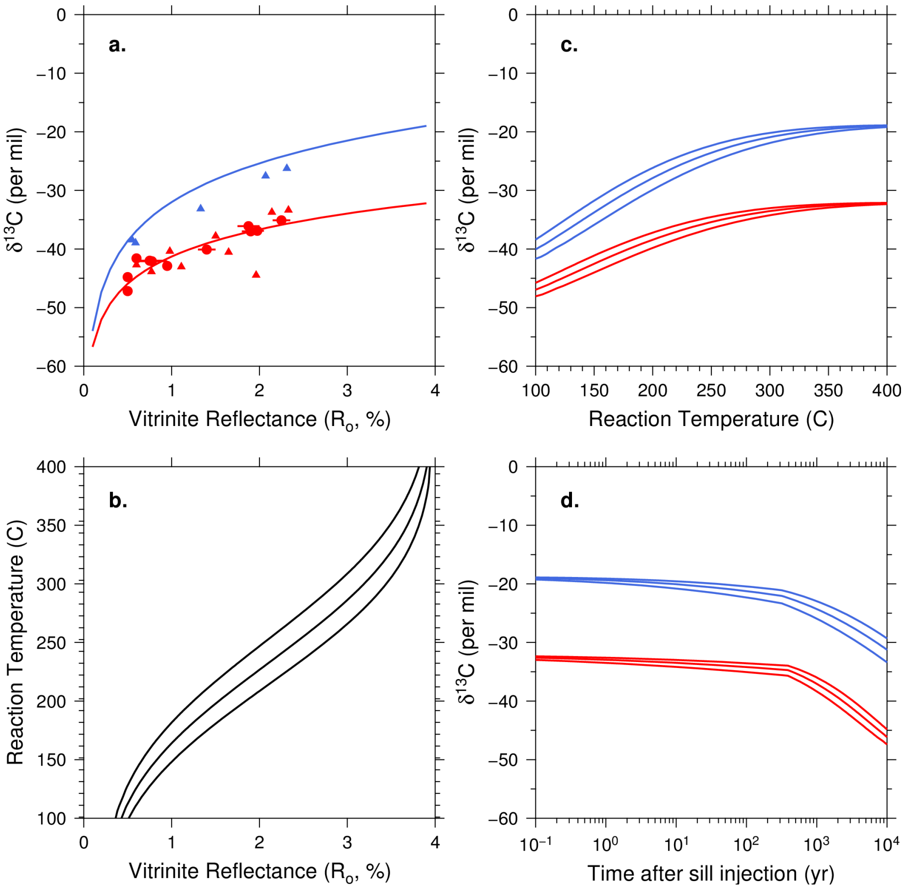

Supplementary Fig. 4. Carbon isotopic compositional variation with kerogen type and maturation temperature.

Blue and red colours indicate gas generation from labile (oil-prone) and refractory (gas-prone) kerogen respectively. **a.** Laboratory data and parameterized relationships between isotopic composition and vitrinite reflectance, a proxy for thermal maturity: circles from[52]; triangles from C. Snape (pers. comm.). Red line is the relationship given in[52] and the blue line is a modified version. **b.** Temperature and vitrinite reflectance at the site of maximum gas generation flux (close to the leading edge of the reaction aureole in Fig. 2), obtained from the coupled thermal-kinetic reaction model for a 200 m thick sill intruded at 5 km. The 3 curves are for heating rates of 0.1, 1 and 10 °C Myr^{-1} (lower to upper). **c.** Composition-temperature relationship from combining the relationships in a. and b. **d.** Corresponding composition-time function: these relationships were used to model combined emissions from the NAIP sill province (Fig. 8).

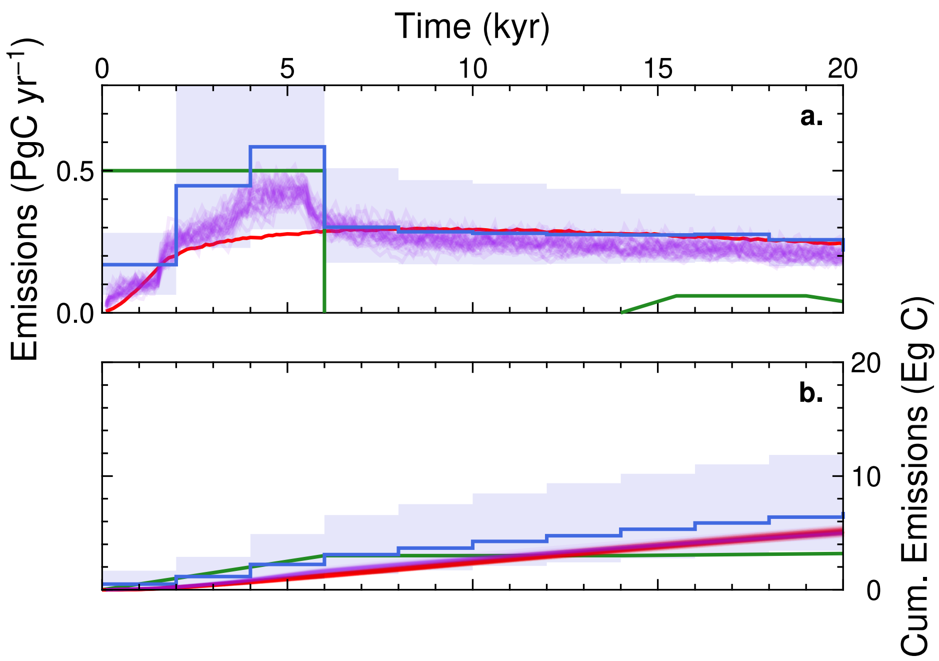

Supplementary Fig. 5. Test of effect of spatial sill distribution on shape of emissions flux peak.

NAIP carbon emissions based on mantle convection model, and comparison with climate-based emissions reconstructions. **a.** Blue line/envelope, emissions flux from joint inverse modelling of oceanic pH and carbon isotope records[5]. Purple cloud: 30 simulated NAIP emissions flux histories calculated in the same way as the red cloud in Fig. 8a, except that the ρ distribution is estimated from the blue line[5] using equation 47 (Fig. 7c,d, grey line). Red line, mean of red cloud in Fig. 8a, which assumed a smooth Gaussian distribution through the ρ measurements (Fig. 7c,d, black line). Green line, emissions from forward modelling of carbon isotope and deep-sea carbonate dissolution records[6,13]. **b.** Cumulative emissions associated with a.

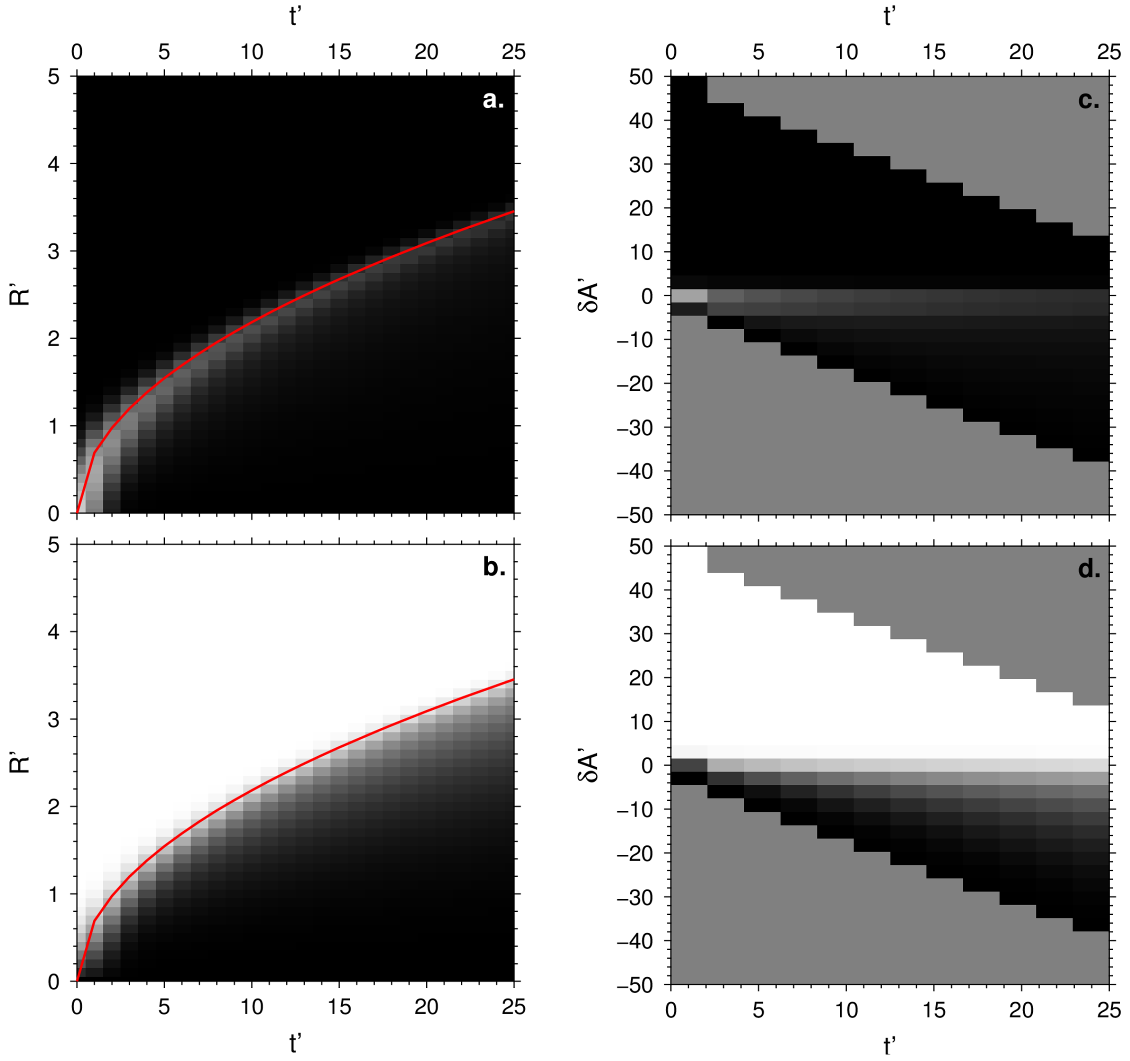

Supplementary Fig. 6. Probability distribution for determining sill radial coordinate in NAIP sill province animations.

a. Probability density function formed from mean plume head temperature plotted as a function of scaled radius $R' = \sqrt{A'/\pi}$ and scaled time t'. Red line shows position of maximum thermal anomaly given by $\sqrt{1.5t'/\pi}$. **b.** Cumulative probability function formed from a. **c.** Probability density function a. re-plotted on axes of deviation in scaled area with respect to marker area, $\delta A'$, against time relative to reference time $1.5A'$. **d.** Cumulative probability function formed from c.; this information was used as a look-up table to determine the sill location radius in Supplementary Movie 1.

Supplementary Tables

Supplementary Table 1. Kinetic parameters for thermal maturation reactions.

Reaction Group	A_E s^{-1}	E_a kJ mol^{-1}	σ kJ mol^{-1}	N_E
RG1 (31)	1.58×10^{13}	208	5	7
RG2 (31)	1.83×10^{18}	279	13	21
RG3 (32)	4.00×10^{10}	242	41	55
RG4 (31)	1.00×10^{13}	230	4	7

Supplementary Table 2. Notation.

Symbol		Definition	Typical Value	Units
d		Distance between sill centres		km
d_{grain}		Density of solid rock grains	2650	kg m^{-3}
d_{host}		Bulk density of host rock	2110	kg m^{-3}
d_{magma}		Density of sill magma	2750	kg m^{-3}
d_{water}		Density of pore water	1030	kg m^{-3}
f	$= 1/\tau_{\mathrm{repeat}}$	Frequency of sill intrusion		yr^{-1}
f_z		Asthenosphere velocity profile function		–
k		Reaction rate		s^{-1}
m_{magma}		Cumulative magmatic C mass from 1 sill		Pg C
m_{therm}		Cumulative thermogenic C mass from 1 sill		Pg C
n		Number of sills		–
p		Power-law exponent for thermogenic C generation		–
q_{magma}		Flux of magmatic C from one sill		Pg C yr^{-1}
q_{therm}		Flux of thermogenic C from one sill		Pg C yr^{-1}
r		Radial coordinate		km
t		Time (relative to start of sill province)		yr
t_0		Time of sill intrusion		yr
Δt	$= t - t_0$	Time relative to sill intrusion		yr
w_{CH4}		Weight% host rock organic C that converts to methane		%
w_{CO2}		Weight% mantle C dissolved in sill magma	0.5	%
z		Depth coordinate		km
A		Sill surface area		km^2
A_{mantle}		Area of mantle plume head		
A_{E}		Frequency factor		km^2

A_{LIP}		Area of LIP sill province		
A_{O}		Overlap between sill aureole footprints		
B		Rate of sediment burial		km yr^{-1}
C		Rate of oil generation		s^{-1}
E_{a}		Activation Energy		kJ mol^{-1}
G		Geothermal gradient	30	K km^{-1}
K		Kerogen ratio (labile:total)	1	–
L		Latent heat of crystallisation	256	kJ kg^{-1}
M_{magma}		Final magmatic C mass		Pg C
M_{therm}		Final thermogenic C mass		Pg C
N		Total number of sills in the province		–
N_E		Number of discrete reactions in E_{a} distribution		–
P		Concentration of reaction product		–
P_0		Initial concentration of reaction product		–
Q		Mantle plume area flux		km^2 yr^{-1}
Q_{prov}		Thermogenic + magmatic C flux from sill province		Pg C yr^{-1}
R		Long radius of plume head		km
R_{g}		Gas constant	8.31451	J K^{-1}mol^{-1}
R_{o}		Vitrinite reflectance		%
S		Maximum sill thickness		m
T		Temperature		K
T_0		Temperature at the seabed	273	K
Y		Total aureole thickness scaled by S		–
Y_{crack}		Total aureole thickness scaled by S, oil cracking reaction		–
Y_{ref}		Total aureole thickness scaled by S, refractory kerogen reaction		–
Z		Depth of sill intrusion		km
Z_O		Vertical overlap between sill aureoles		km
β		Sill radial thickness variation parameter		–
δ		Standard deviation of mantle temperature pulse	40	kyr
δ^{13}C		Carbon isotopic composition of emissions		–
$\delta^{13}_{\mathrm{lab}}\mathrm{C}$		Carbon isotopic composition of emissions from labile kerogen		–
$\delta^{13}_{\mathrm{ref}}\mathrm{C}$		Carbon isotopic composition of emissions from refractory kerogen		–
γ		Plume head aspect ratio		–
ϕ		Sediment porosity		–
ϕ_0		Initial sediment porosity	0.61	–
φ		Aureole overlap volume fraction		–
κ		Sediment thermal diffusivity	10^{-6}	m^2 s^{-1}
λ		Compaction length scale	2	km
ρ		Sill area density		km^{-2}

σ		Standard deviation of activation energies		kJ mol^{-1}
τ_{cond}		Sill conductive cooling time		yr
τ_{decay}		Sill thermogenic emissions decay time		yr
τ_{repeat}	$= 1/f$	Sill repeat time period		yr
τ_{solid}		Sill solidification time		yr
τ^*	$= \tau_{decay}/\tau_{cond}$	Sill thermogenic emissions decay time		yr
Γ		Sill province areal expansion function		–
Π		Normal probability distribution		–
