# Supplementary material for: Large Igneous Province thermogenic greenhouse gas flux could have initiated Paleocene-Eocene Thermal Maximum climate change
Source: Nat Commun. 2019 Dec 5;10:5547. doi: 10.1038/s41467-019-12957-1 (PMC6895149; doi:10.1038/s41467-019-12957-1)
Supplement: Supplementary file 3 — Description of Additional Supplementary Files [file 41467_2019_12957_MOESM3_ESM.pdf]

Description of Additional Supplementary Files

File name: Supplementary Movie 1

Description: Model evolution of NAIP sill province. Animation to show how igneous sills progressively intrude beneath the deep basin chain at increasing distances from the plume centre as a hot mantle ring beneath sweeps outward beneath the plate. Red circles show sill-vent complexes currently emitting carbon, shade of red proportional to emissions flux, size of circle proportional to final cumulative emissions (horizontal scale is exaggerated). Older sill-vent systems no longer emitting carbon are coloured grey. Pale blue ellipses show position of the thermal anomaly in the sub-plate convecting mantle which generates the magma that intrudes as sills, as well as transient uplift of Earth's surface: heavy line marks the peak thermal anomaly; thin line marks 5% of the peak thermal anomaly. Time is relative to the time when the peak thermal anomaly lies at the plume centre[38]. Pie chart shows proportions of sills venting direct to atmosphere (orange), into shallow water (< 1 km water depth, pale blue) and into deep water (> 1 km water depth, dark blue). Figs 8g–j are snapshots from this model at times of 2, 10, 20 and 50 thousand years. Figs 3a–f show flux, mass and composition of combined carbon emissions from many stochastic variations of the model in this movie.
